# Introducing the Sanguis-Filum for Detection of Gastric Mucosal Lesions Prior to Endoscopy: A Study Protocol

**DOI:** 10.3390/diagnostics12051160

**Published:** 2022-05-07

**Authors:** Violet Kayamba, Paul Kelly

**Affiliations:** 1 Tropical Gastroenterology and Nutrition Group, University of Zambia School of Medicine, Lusaka P.O. Box 50398, Zambia; m.p.kelly@qmul.ac.uk; 2 Blizard Institute, Barts & The London School of Medicine and Dentistry, Queen Mary University of London, 4 Newark Street, London E1 2AT, UK

**Keywords:** gastric cancer, diagnosis, guaiacum, Sanguis-filum

## Abstract

Early diagnosis of gastric cancer (GC) is compromised by a lack of specific signs to enable identification of affected individuals. We designed the Sanguis-filum (S-filum) as a simple bedside tool that could be used to detect the presence of gastric mucosal lesions prior to endoscopy. We previously published evidence that at a sensitivity of 91%, the presence of free blood in the stomach was associated with mucosal lesions. The S-filum is made of an inert but absorbent string coiled up in a gelatin capsule (Capsuline, FL, USA), which can be swallowed and the string retrieved to test for free blood. Preliminary testing of the S-filum was successfully conducted on healthy volunteers. We now intend to test it on actual patients, comparing the results to oesophagogastroduodenoscopy (OGD) findings. This will enable us to determine the diagnostic accuracy of the S-filum at detecting GC and other mucosal lesions. The S-filum as a bedside tool has the potential to assist healthcare providers to identify individuals likely to have early gastric mucosal lesions and requiring OGD examination. The S-filum could, in the long run, facilitate population-wide screening for early GC prior to endoscopy.

## 1. Introduction

Gastric cancer (GC) is one of the leading causes of cancer related deaths in the world. It is the fourth most common cancer with about 1,089,103 new cases reported in 2020 [1]. In Africa, it is the 9th most common cancer type and 32,402 cases were reported in 2020 [1]. The highest age standardised incident rates for GC are in the Eastern Asian population at 32.5 per 100,000 in men and 13.2 per 100,000 in women. By contrast, the rates in the North American population are 5.4 per 100,000 in men and 3.1 per 100,000 in women. As a continent, the African population has the lowest age standardised incident rates of 4.5 to 4.9 per 100,000 in men and 3.8 to 4.2 per 100,000 in women [1].

Average survival rates for GC are very poor, being at 32% in the United States, and 26% in Europe [2]. The survival rates are higher in countries such as Korea [3] and Japan, [4] as they have early case detection programs. There is documented evidence of a modest improvement of GC outcomes over the past decades, being better in younger age groups [5]. The mortality-to-incidence ratio (MIR), which measures deaths in relation to disease occurrence for GC, is lowest in countries with a better human development index and higher health expenditures [6]. Africa has the highest mortality MIR of 0.91, necessitating an urgent exploration of mitigating strategies. Molecular and histological sub-types influence GC prognosis [7], but the most significant factor is stage of disease at first diagnosis.

Early GC is predominantly asymptomatic, which presents a diagnostic challenge. It is difficult for healthcare providers to know which individuals (with non-specific symptoms) need endoscopic examination to check for the presence of early GC. Image-enhanced upper gastrointestinal endoscopy is the gold standard for early GC diagnosis [8], but it is expensive and its availability is scanty [9]. Even when available, it can only be offered to a limited number of individuals at a time, and the lack of specific GC symptoms means that some patients will end up being missed, or their disease diagnosed late resulting in poor treatment outcomes.

There is evidence that late GC diagnosis in Zambia is as a result of late referral for endoscopy and not necessarily delayed patient presentation [10]. A major contributor to late requests for endoscopy is that health care providers in primary and secondary settings find it difficult to identify patients with a high likelihood of GC. Alarm symptoms of GC such as weight loss, microcytic anaemia, vomiting (bloody or non-bloody), or abdominal swelling usually become apparent in advanced disease, and at a population level, their predictive values are generally low [11,12]. Abdominal pain commonly occurs in GC, but it is non-specific and also a very common symptom of benign gastric disease [13] and therefore not a discriminatory symptom.

Image enhancing endoscopic techniques improve the detection of early GC, but the usefulness of many such techniques is under discussion [14]. Most of these techniques are not available in low-resource settings. Currently, there is no simple tool or method that can be used in low-resource settings to recognise patients most likely to have early GC and who are in need of endoscopic evaluation. Preliminary work demonstrated that the presence of free blood in the stomach was associated with GC [15]. In that study, gastric juice was collected during endoscopy and the presence of free blood determined using urinalysis test strips. The presence of free blood in gastric juice was significantly associated with GC and other mucosal lesions. Building on this, we designed a simple tool that could be used in a wide variety of settings. We called it the Sanguis-filum or S-filum (two Latin words meaning blood and string).

Our overall vision for the S-filum is for it to be adopted as a triage tool in primary care facilities. This will enable health care providers to identify patients most likely to have gastric mucosal lesions and in need for endoscopic examination. As the S-filum is very cheap to produce, we envision that it could also be used as a screening tool at population level, a strategy that is currently impossible in many settings.

The primary objective of this study will be to determine the diagnostic accuracy of the S-filum for detection of early GC. Using endoscopic diagnosis as the gold standard, we will compute the specificity, sensitivity, negative and positive predictive values for the S-filum. The secondary objective will be to determine how well the S-filum will detect other gastric mucosal lesions such as ulcers and erosive gastritis.

The study protocol was approved by the University of Zambia Biomedical Research Ethics Committee reference number 2446–2021.

## 2. Experimental Design and Materials

### 2.1. Description of the S-Filum

**Figure 1:**
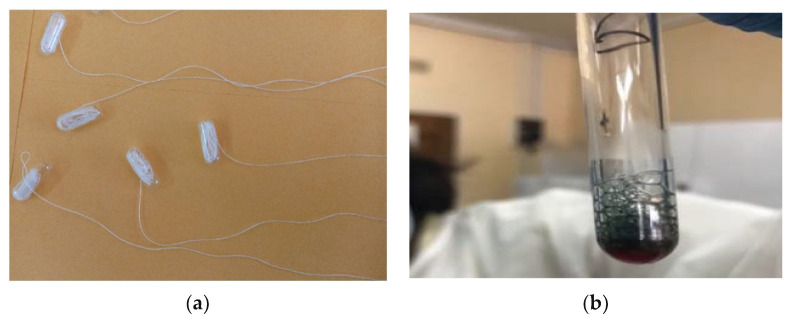
Panel (**a**) Images of the S-filum; (**b**) an example of the blue colour change occurring on mixing dissolved guaiacum powered, hydrogen peroxide and a solution containing blood.

The S-filum (Figure 1a) is an inert but absorbent string coiled up in a gelatin capsule (Capsuline, FL, USA). The capsules were made using 100% pharmaceutical grade gelatin derived from only beef, without any additives or contaminants. They are Kosher and Halal certified and also gluten free.

### 2.2. Study Design

This will be a cross sectional diagnostic study [16]. It will be conducted in the endoscopy unit of the University Teaching Hospital, Lusaka. Patients presenting for diagnostic oesophagogastroduodenoscopy (OGD) will be considered for study participation. Included will be patients above the age of 18 years and those having given informed written consent to participate. These patients will present for OGD after a fasting for at least eight hours. We will exclude patients with dysphagia or those presenting with a history of gastrointestinal bleeding (haematemesis or melaena) or referred because of a positive faecal occult blood test result.

**Figure 2:**
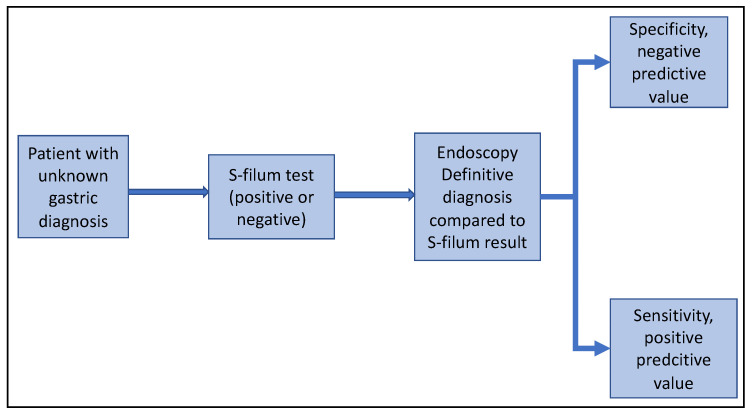
Summary of patient enrollment and determination of validity of the S-filum.

The S-filum testing will be applied on consenting patients before OGD and the result recorded as either positive or negative, Figure 2. The tests whose colour change is not very clear will be deemed classified as indeterminate. To minimize bias, the investigator applying and interpreting the S-filum result will not be aware of the indication for OGD. Blinded to the S-filum result, the endoscopist will conduct the procedure in accordance with standard of care and the appropriate diagnosis recorded. Upon entering the stomach during OGD, a sample of gastric juice will be drawn for pH testing using commercial test strips as published previously [17]. Using OGD findings as the gold standard, the diagnostic accuracy of the S-filum will be computed. Computed from these results will be the sensitivity, specificity, positive predictive value, and negative predictive value, and receiver operating characteristic curve will be plotted.

### 2.3. Sample Size Calculation

In a preliminary study to test the concept of using blood collected during endoscopy for diagnosis, we found positive results in 56% of those without mucosal lesions and 73% among those with lesions including gastric cancer and benign ulcers [15]. Using these estimates with a power of 90%, and a two-sided alpha of 0.05, we would need a total of 352 participants, (176 with and 176 with free blood in the lumen). To allow for drop outs, we will enrol 400 study participants. 

### 2.4. Data Collection, Management and Analysis

Demographic characteristics for the study participants will be collected using interviewer administered questionnaires. Results for the S-filum will be recorded as either positive, negative or indeterminate. Endoscopy reports will be produced in accordance with the standard of care.

Data will be double entered into Epi Data and checked for accuracy. It will then be analysed using STATA 15 (Stata Corp, College Station, TX, USA). 

Continuous variables will be summarized using mean and standard deviation for normally distributed variables and medians with interquartile ranges for non-normally distributed variables. Two-way analyses will be employed to look for associations between the GC and the exposure of interest considering potential confounders. Sensitivity, specificity and predictive values calculated. The Fisher’s exact test will be used for categorical variables, and the Kruskal-Wallis rank tests for continuous variables. Odds ratios with 95% confidence intervals will be derived and *p* value less than 0.05 will be considered statistically significant. 

## 3. Preliminary Work Carried Out to Design the S-Filum

To demonstrate the utility of guaiacum powder at detecting the presence of blood and to investigate pH dependency of this reaction, we used guaiacum powder, 99% ethanol, hydrogen peroxide, and water. In a serious of experiments, we were able to confirm that mixing dissolved guaiacum powder, hydrogen peroxide and blood resulted in a colour change from brown to blue, Figure 1b. This occurred even after dilution with water, as illustrated in Table 1 (colour change was seen in tubes 5 and 6). Using 1.0 M hydrochloric acid (HCl), the same acid as found in the stomach, we prepared various solutions with pH ranging from 0 to 7. To 200 μL of each of these solutions, we added different amounts of blood (1 μL, 5 μL,10 μL, 20 μL, 30 μL, and 40 μL) and the same amounts of dissolved guaiacum powder and hydrogen peroxide. Colour changes were observed in each tube, as shown in Table 2. 

The experiments demonstrated the expected colour changes at pH 3 to 7 for blood concentrations of 5 μL or greater in 200 μL solution. A blood concentration of 10 μL in 200 μL solution was required at pH of 2 and 40 μL at pH 1. Therefore, the sensitivity of the test would be reduced at lower pH values. To counter this limitation, we will collect gastric juice endoscopically in all the patients and measure the pH. We will then factor in the pH when analysing the results. Further work may be needed to determine the value of ingestion of a neutralising buffer prior to the S-filum test.

Next, we explored the effect of pH on capsule dissolution. Using the solutions prepared above, we found that the capsules dissolved completely within 31 min at all pH levels tested (Table 3). 

Lastly, we endeavoured to determine whether the string was able to absorb enough liquid to facilitate testing. We therefore dipped strings in the various solutions prepared above. In all cases, the colour change of the string was consistent with that of the solution it was dipped into.

## 4. Results from the S-Filum Testing on Volunteers

With the information gathered from the above preliminary experiments, we tested the S-filum on two volunteers within our laboratory. The volunteers swallowed the capsule with a small amount of water (50–70 mL), and the proximal end of the string was strapped to the cheek. After 35 min, the string was pulled out and tested for traces of free blood by placing it into a mixture of guaiacum powder dissolved in 99% alcohol and hydrogen peroxide. Both tests were negative for blood. The volunteers tolerated the procedure very well reporting no significant discomfort apart from the sensation of a string in the throat. 

## 5. Expected Results from This Study

With the S-filum testing, we expect to categorise the patients with either having free blood in the stomach (positive) or no blood (negative), and a few might have indeterminate results. Endoscopy findings will show either a normal stomach or evidence of mucosal lesions such as GC, ulceration, erosions, gastritis, or polyps. The comparison of these results will enable the determination of the diagnostic accuracy of the S-filum.

In addition, some patients will also have lesions in either the oesophagus or the duodenum. The diagnostic accuracy for these non-gastric lesions will be analysed separately as they do not constitute the main focus of this study.

## 6. Discussion

We designed the S-filum as a novel tool that has the potential to assist healthcare providers in deciding which patients to send for endoscopy. If accurate, this will be of value in rural settings where access to endoscopy is limited. It is a simple bedside tool that tests for the presence of free blood in the stomach. Patients with positive results will require an endoscopy. The S-filum can be used by medical personnel with basic training and will therefore, be easily applicable in low-resource settings. With this strategy, it might be possible to diagnose GC earlier, ultimately improving patient outcomes. The S-filum also has the potential for use as a screening tool in seemingly healthy populations.

A lot of research has been done to identify biomarkers or clinical strategies that might facilitate early GC diagnosis. These include molecular markers measured in blood, urine, or gastric juice [18]. Molecular markers require trained personnel, sophisticated equipment, and reagents that are not readily available in low-resource settings. These, therefore, cannot be rolled out by many health care systems.

Periodic endoscopic surveillance is another modality for early GC detection [19], but it is very expensive and requires a steady supply of water, electricity, and well-trained personnel to conduct the procedures safely and accurately. Population-based endoscopic screening is carried out in countries with high GC rates such as Korea and Japan, as early detection of GC using screening programmes improves its outcome [20,21]. There is also evidence that countries with GC screening programmes have higher rates of early cancer detection than those that do not [22]. However, these screening programmes are very expensive and impractical in some settings, as they require repeated endoscopic evaluations on a high number of people. For example, the National Cancer Control Committee of Korea recommends that individuals over the age 40 years undergo GC screening via either the upper gastrointestinal series or endoscopy every 2 years [23]. 

Many other health care systems are unable to support such an elaborate programme, and even those that can afford it might not have enough GC cases to justify it. Population coverage of these screening programmes can be a challenge if not well organized. In Chile, the GC programme was reported to have coverage as low as 14% [24]. The programme in Korea, however, is well organized, and it has been shown to be available to patients of all socio-economic classes [25]. Other non-invasive methods of identifying individuals with increased risk of having GC have been considered. The *H. pylori* test-and-treat strategy has been proposed for populations with high GC incidence [26]. 

In Zambia, the use of *H. pylori* antibodies to identify these high-risk individuals would not be discriminating enough, as over 80% of the population is positive [27,28]. However, the rationale of using antibody titers and pepsinogens could be helpful, but it is yet to be validated [29]. There is evidence from a local study that using the GastroPanel, which includes testing for *Helicobacter pylori* (*H. pylori*), pepsinogens, and gastrin-17, poorly predicts gastric premalignancy in HIV-infected Zambian patients [17]. It was, however, shown to be helpful at diagnosing chronic atrophic gastritis in other populations [30]. 

Faecal occult blood testing (either using immunochemical or guaiac-based testing) is a well-established strategy for colorectal cancer screening, [31] but not for GC. There is evidence that FOB cannot be recommended for screening upper gastrointestinal cancers [32,33]. The need for a simple tool such as the S-filum is therefore well justified. Documented concerns of medication such as oral iron or ascorbic acid affecting the guaiac reaction [34] will not apply to the S-filum. It will be conducted on fasted patients and it directly evaluates the gastric juice. We must clearly point out that the S-filum testing is not intended to substitute endoscopy, but rather serve as a screening tool to assist healthcare providers in deciding which patients to priorities for the procedure, when access is limited. 

The major strength of this study design is that the endoscopist will be blinded to the S-filum results at the time of examination. This will allow for independent assessment producing more reliable results. In addition, we have already tried out the S-filum on volunteers and it was well received. However, patients will experience minor discomfort due to feeling a string in the throat while the test is being conducted. Otherwise, swallowing a capsule with some water is painless. We will not be giving the participants any investigative agents. The testing will be done with the close supervision of trained nurses and/or doctors. Withdrawal of the string causes further minor discomfort but is painless.

A limitation of the S-filum is the requirement to use water to swallow the capsule. This might result in dilution of the gastric juice. We however do not think this will significantly affect the sensitivity of the test as our experiments showed a colour change after dilution. We have also taken into consideration the possible effects of pH, and it will be factored in during data interpretation and analysis.

In conclusion, this study will determine the diagnostic accuracy of the S-filum foe r the diagnosis of early GC and other mucosal lesions. The S-filum has considerable potential as a screening tool for prioritising patients for OGD. It could make it possible to roll out population-wide screening for GC and mucosal lesions. 

## Figures and Tables

**Table 1:** Test to demonstrate guaiacum powder colour change when in contact with hydrogen peroxide and blood.

	H_2_0	H_2_0_2_	Guaiacum	Blood	Result
Tube 1	+	+	–	–	No colour change
Tube 2	–	+	–	+	No colour change
Tube 3	–	+	+	–	No colour change
Tube 4	–	–	+	+	No colour change
Tube 5	–	+	+	+	Colour change to blue
Tube 6	+	+	+	+	Colour change to blue

The (+) means that the substance was added, while the (–) means that the substance was not added.

**Table 2:** Experiments to determine the effect of pH on colour changes.

pH of the Solution	Volume of Blood Added in μL					
	1	5	10	20	30	40
pH 0	–	–	–	–	–	–
pH 1	–	–	–	–	–	+
pH 2	–	–	+	+	+	+
pH 3	–	+	+	+	+	+
pH 4	–	+	+	+	+	+
pH 5	–	+	+	+	+	+
pH 6	–	+	+	+	+	+
pH 7	–	+	+	+	+	+

The (+) denotes colour change while the (–) denotes no colour change.

**Table 3:** Time taken for the capsules to completely dissolve at various pH values.

Tube (pH)	Dissolving Time (min)
0	23
1	25
2	29
3	29
4	30
5	31
6	29
7	28
